# Practical Notes and Formulæ

**Published:** 1885-11-20

**Authors:** 


### PRACTICAL NOTES AND FORMULÆ.

**Ileo-colitis.**—A case of ileo-colitis, occurring in a boy, æt. 10, was treated by Professor Bartholow, as follows: Cut off starchy and fatty substances of his diet, but give skimmed milk and beef juice, also the white of eggs, if this agrees with him; and three times a day give—

℞. Bismuthi subcarb........................gr. xv,
Acidi carbolici........................gr. ¼. M.

**Alopecia.**—For alopecia (College and Clinical Record), Professor Gross, among other things, advises that the hair be shingled, then head cleansed with—

℞. Saponis viridis........................℥ ij,

Aquæ Cologniensis........................f ℥ iv. M.

Sig. As a wash every third night; then use several times daily—

℞. Tinct. cantharid........................f ʒ iss,

Tinct. capsici........................gtt. xx,

Glycerini........................f ʒ ss,

Aquæ Cologn., q. s. ad........................f ℥ i. M.

**To Relieve the Violent Pains of Lead Colic.**—Bartholow, in Medical World:

℞. Aluminis........................ʒ ij,

Acid. sulph. dil........................ʒ j,

Syr. limonis........................℥ j,

Aquæ........................℥ iij. M.

Sig. A tablespoonful every hour or two.

**Angostura Bitters.**—Medical World: Take of—

℞. Angostura bark........................oz. 4

Chamomile flowers........................oz. 1

Cardamon seeds........................oz. ¼

Cinnamon bark........................oz. ¼

Orange peel........................oz. 1

Raisins........................℔. 1

Proof spirits........................gal. 2½

Macerate for a month, press and filter.

**Pill for Syphilis.**—This (Medical World) is Prof. Berkeley Hill's formula. It renders excellent service:

℞. Hydrargyri bicyanidi........................gr. j
Quiniæ sulphatis........................gr. xx,
Ext. gentianæ comp........................gr. xxx. M.

Sig. Divide into 20 pills; one to be taken two or three times a day.

**For Sexual Debility.**—Dr. L. Lewis, in Medical World :

℞. Phospori....................................gr. I
Ext. nucis vomicæ.............................gr. x
Ext. cannabis Indicæ..........................gr. v. M.

Divide into 20 pills. Sig. One night and morning.

**For Falling Hair.**—Dr. W. P. Grate (in New England Medical Monthly) says : The following recipe will prove of interest to those of your readers who are troubled with falling hair. It is most excellent, and I can recommend it :

℞. Bay rum..................................℥ ij,

Glycerine...................................℥ ij,

Tincture of cantharides.....................ʒ j,

Oil of bergamot.............................ʒ ss,

Puinia sulph................................grs. x,

Aqua........................................℥ iv. M.

Use every morning for dressing the hair, and discard all oil and grease.

**Villate's Mixture in Gleet.**—New England Medical Monthly gives the formula as follows, as a injection :

℞. Liquor plumbi sub acetas.................. ʒ j,

Crystals of the sulphate of zinc } aa...........℥ ss,

Cupri sulphas, }

White wine vinegar..........................℥ vjss. M.

One part of this to ten of water is said to be very efficacious.

**Freckle Wash.**—The American Pharmacist gives this :

℞. Tr. benzoin.............................. I oz.

Muriate ammonia............................120 grs.

Rose water................................ 7 ozs. M.

To be applied at night.

**Hair Dye.**—A good hair dye (American Pharmacist) said to be like Buckingham's may be prepared as follows :

℞. Nitrate of silver.........................I oz.

Nitrate of copper...........................I dr.

Distilled water.............................8 oz.

Water of ammonia............................q. s.

Dissolve the metallic salts in distilled water, and add ammonia until the liquid becomes a clear hue. Apply as usual, and exposure to the rays of the sun will turn the hair black.

**A Calf Weaner** has been patented by Edward P. Henry, of Eagle Rock, Idaho Ter. It is for attachment on the nose of a calf, and consists of two plates pivoted to each other at the edges, each plate having a curved prong at the upper inner corner, preventing the calf from sucking, but permitting it to eat grass.—*Scientific American.*

